# Supplementary material for: Quantification of amyloid fibril polymorphism by nano-morphometry reveals the individuality of filament assembly
Source: Commun Chem. 2020 Sep 11;3:125. doi: 10.1038/s42004-020-00372-3 (PMC9814634; doi:10.1038/s42004-020-00372-3)
Supplement: Supplementary file 1 — Supplementary Information [file 42004_2020_372_MOESM1_ESM.pdf]

Quantification of amyloid fibril polymorphism by nano-morphometry reveals the individuality of filament assembly

Supplementary Figures

Liam D. Aubrey [1†], Ben J. F. Blakeman [1†], Liisa Lutter [1], Christopher J. Serpell [2], Mick F. Tuite [1], Louise C. Serpell [3], Wei-Feng Xue [1*]

[1] Kent Fungal Group, School of Biosciences, University of Kent, CT2 7NJ, Canterbury, UK

[2] School of Physical Sciences, University of Kent, CT2 7NH, Canterbury, UK

[3] Sussex Neuroscience, School of Life Sciences, University of Sussex, BN1 9QG, Falmer, Brighton, UK

[†] Authors contributed equally to this study

* Correspondence to: W.F.Xue@kent.ac.uk; Tel +44-(0)1227 824821

HYFNIF

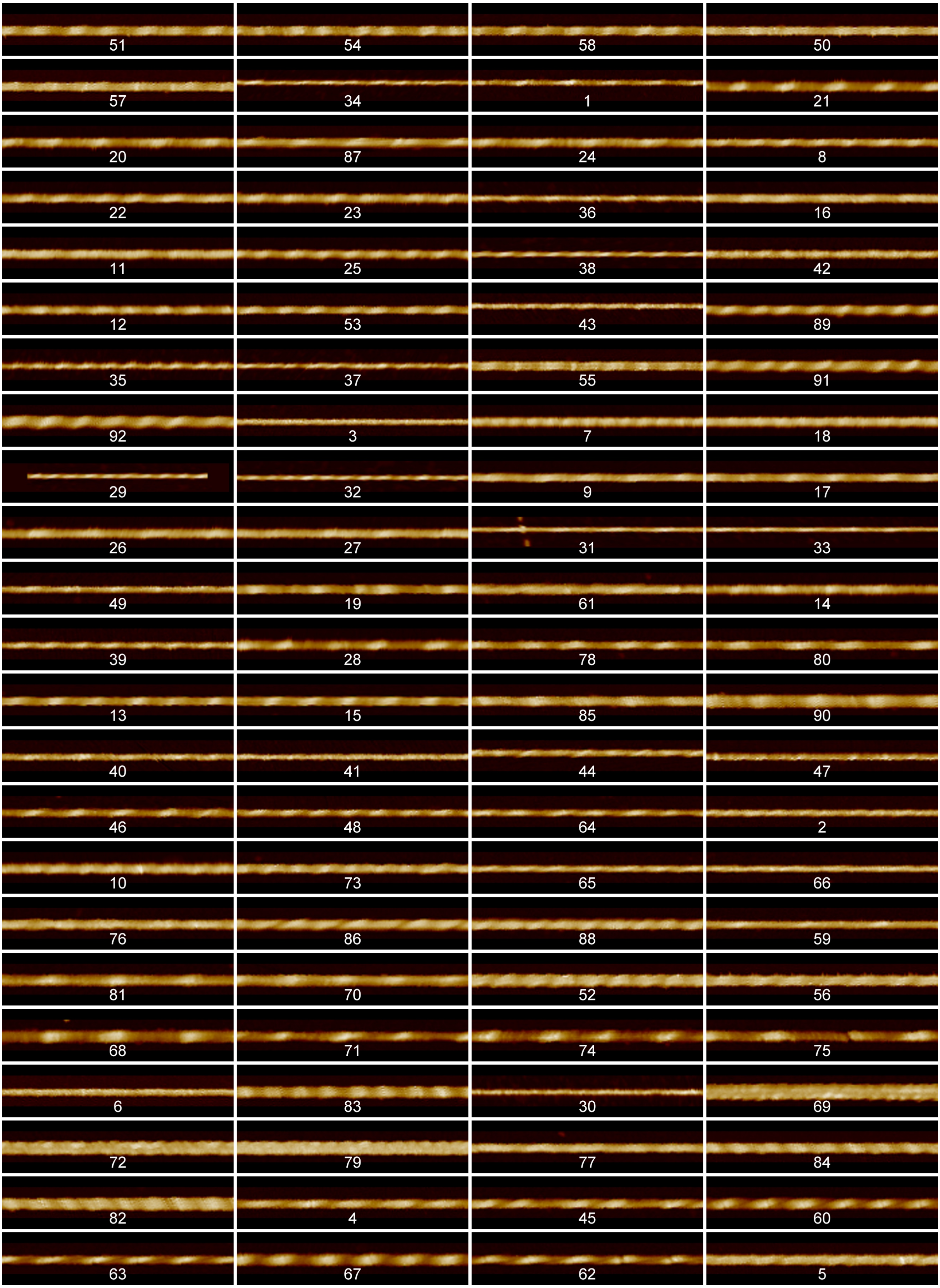
51
54
58
50
57
34
1
21
20
87
24
8
22
23
36
16
11
25
38
42
12
53
43
89
35
37
55
91
92
3
7
18
29
32
9
17
26
27
31
33
49
19
61
14
39
28
78
80
13
15
85
90
40
41
44
47
46
48
64
2
10
73
65
66
76
86
88
59
81
70
52
56
68
71
74
75
6
83
30
69
72
79
77
84
82
4
45
60
63
67
62
5

RVFNIM

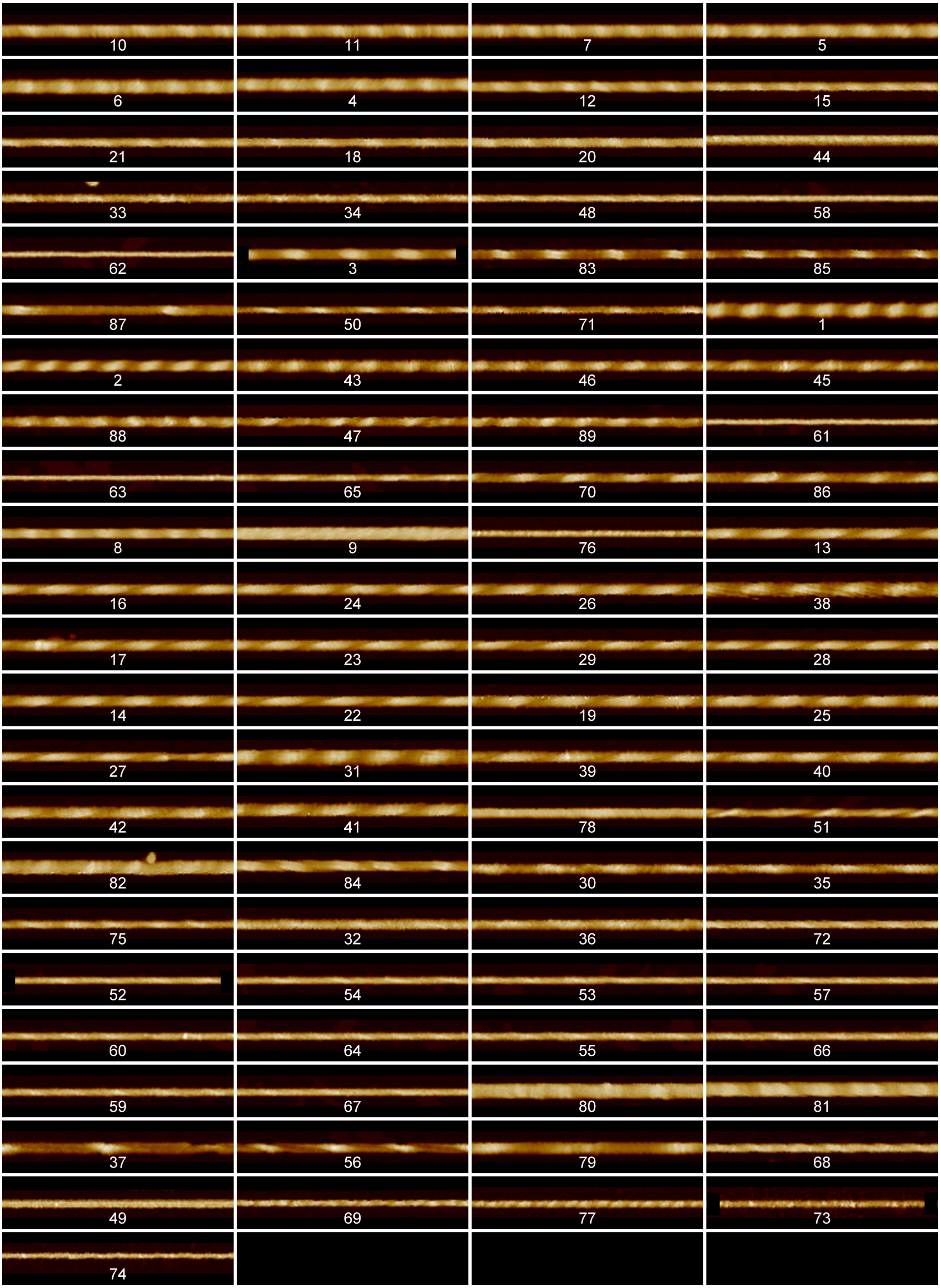
10 11 7 5
6 4 12 15
21 18 20 44
33 34 48 58
62 3 83 85
87 50 71 1
2 43 46 45
88 47 89 61
63 65 70 86
8 9 76 13
16 24 26 38
17 23 29 28
14 22 19 25
27 31 39 40
42 41 78 51
82 84 30 35
75 32 36 72
52 54 53 57
60 64 55 66
59 67 80 81
37 56 79 68
49 69 77 73
74

VIYKI

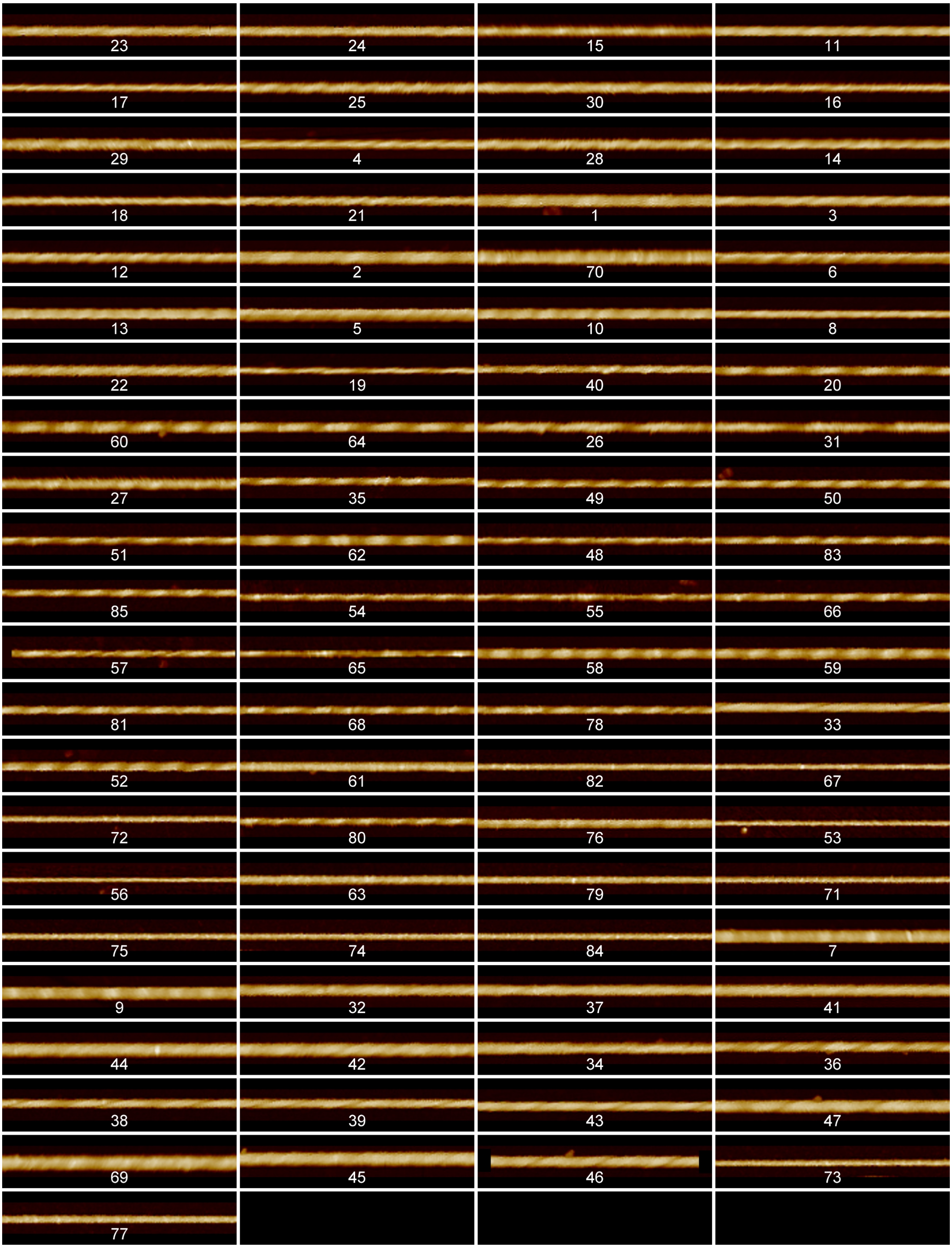
23
24
15
11
17
25
30
16
29
4
28
14
18
21
1
3
12
2
70
6
13
5
10
8
22
19
40
20
60
64
26
31
27
35
49
50
51
62
48
83
85
54
55
66
57
65
58
59
81
68
78
33
52
61
82
67
72
80
76
53
56
63
79
71
75
74
84
7
9
32
37
41
44
42
34
36
38
39
43
47
69
45
46
73
77

Supplementary Figure 1: AFM image data sets for all of the individually traced fibrils. All fibrils displayed here were straightened and they were cropped to 500 nm segments for visualisation here if the contour length is longer than 500 nm. No further processing occurred. The fibrils are shown with their individual index numbers (see **Supplementary Data**), and are arranged by similarity (see **Figure 7** and **Supplementary Figure 6**).

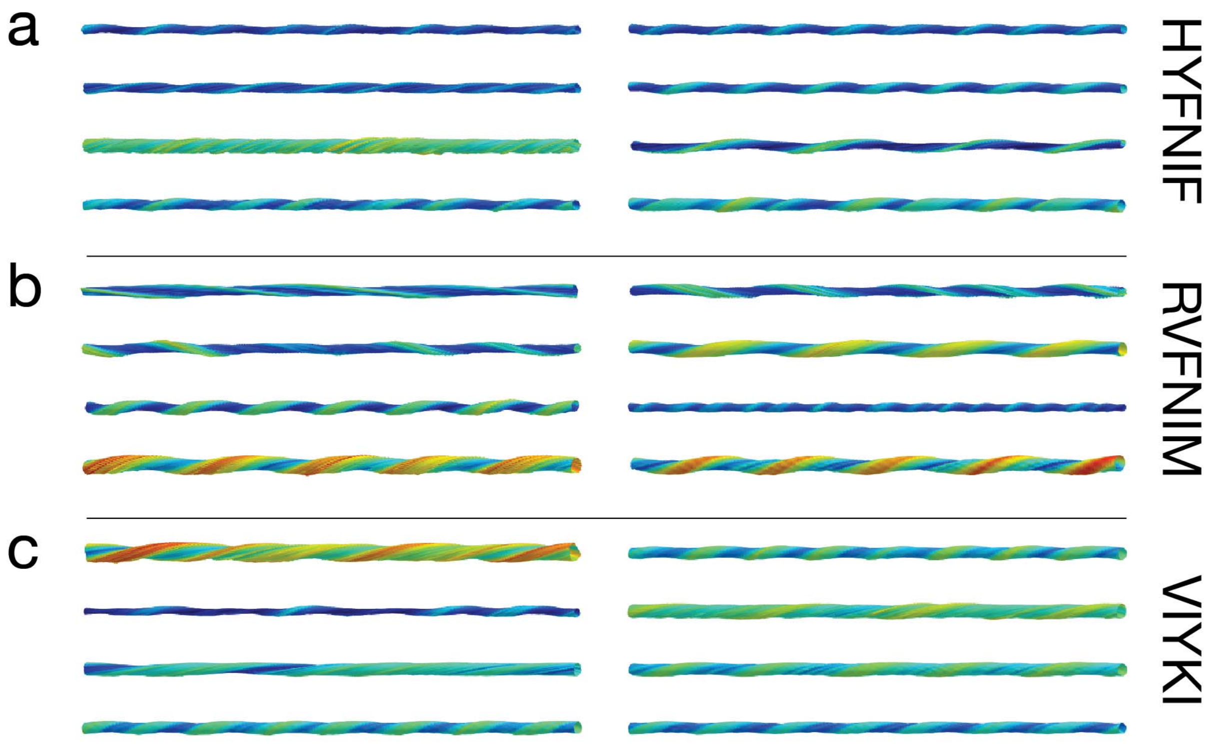

Supplementary Figure 2: 3D models of Waltz peptide assemblies. A selection of typical reconstructed fibril 3D models is shown. Each fibril in the three data sets was reconstructed as a 3D models using information and 3D coordinates extracted directly from the AFM data. The average cross-sectional area and the helical symmetry was determined from the generation of each 3D model. Models for 8 fibrils from each assembly reaction, HYFNIF (a), RVFNIM (b) and VIYKI (c) are displayed here. The models displayed here represent the same fibrils displayed in the image data in **Figure 3**. All of the models are shown with identical scale and the colour scale represent the local radius to the screw axis for visualisation. All of the models (around 90 fibrils for each data set) are displayed in **Figure 4**.

HYFNIF

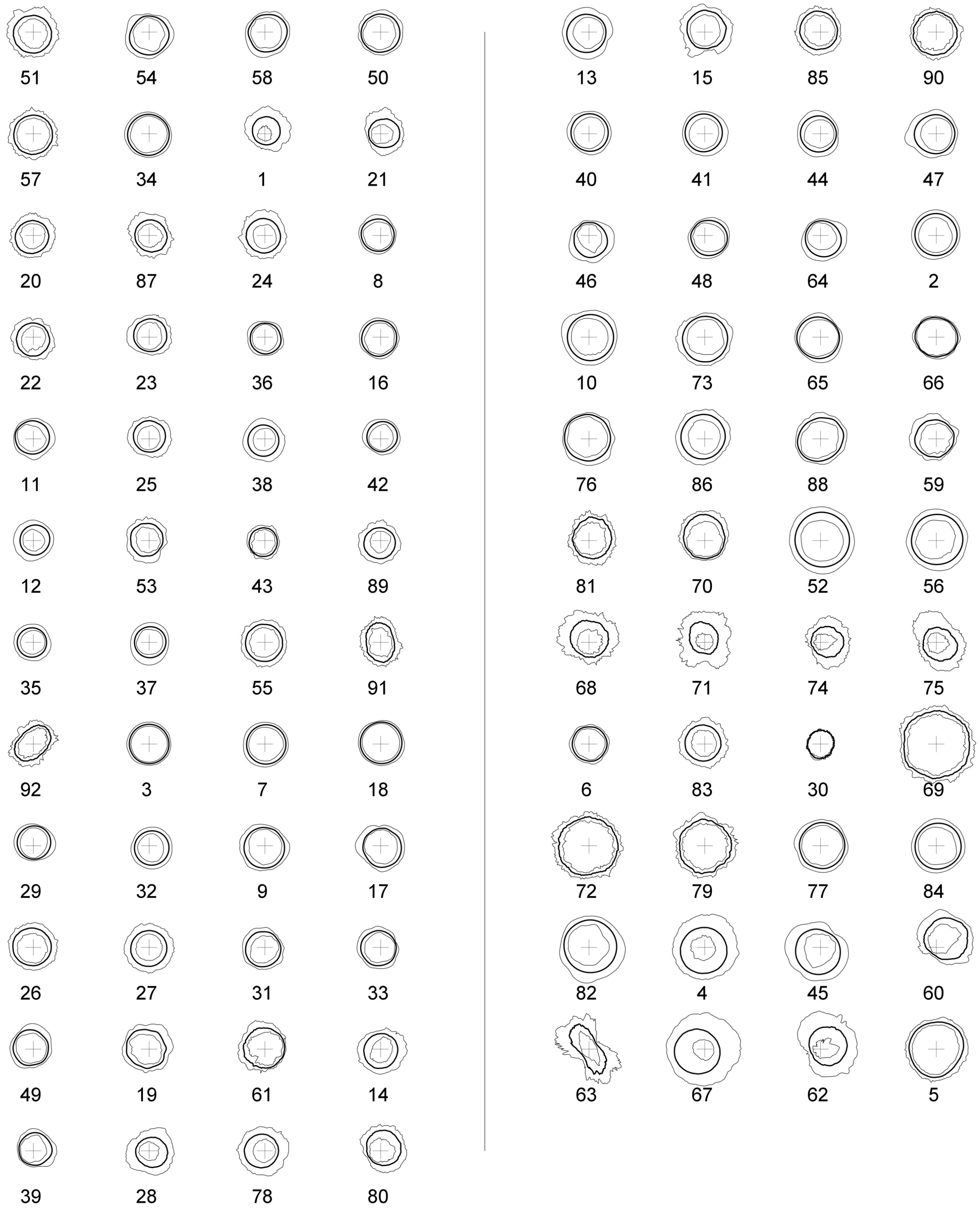
51 54 58 50
57 34 1 21
20 87 24 8
22 23 36 16
11 25 38 42
12 53 43 89
35 37 55 91
92 3 7 18
29 32 9 17
26 27 31 33
49 19 61 14
39 28 78 80
13 15 85 90
40 41 44 47
46 48 64 2
10 73 65 66
76 86 88 59
81 70 52 56
68 71 74 75
6 83 30 69
72 79 77 84
82 4 45 60
63 67 62 5

RVFNIM

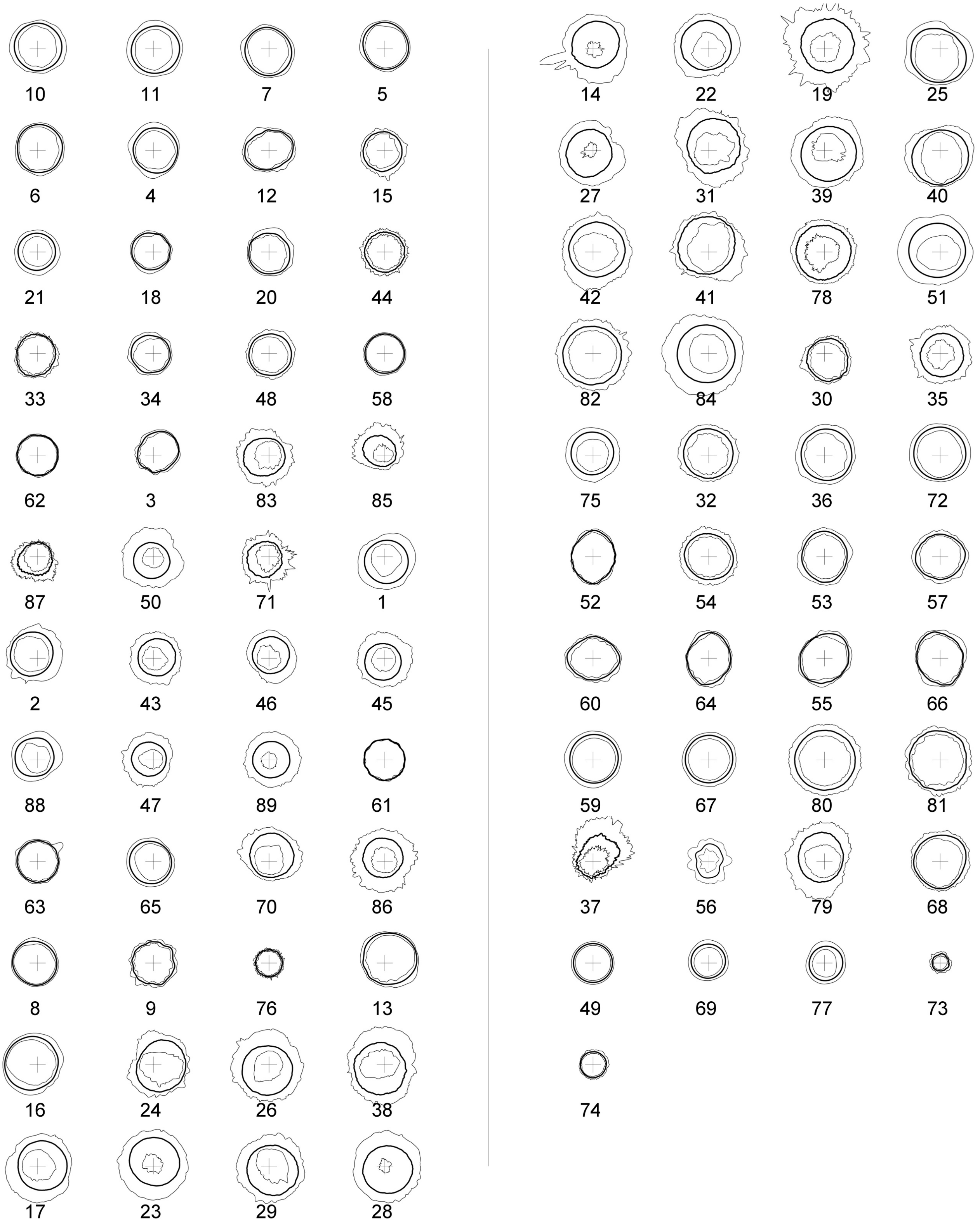
10 11 7 5
6 4 12 15
21 18 20 44
33 34 48 58
62 3 83 85
87 50 71 1
2 43 46 45
88 47 89 61
63 65 70 86
8 9 76 13
16 24 26 38
17 23 29 28
14 22 19 25
27 31 39 40
42 41 78 51
82 84 30 35
75 32 36 72
52 54 53 57
60 64 55 66
59 67 80 81
37 56 79 68
49 69 77 73
74

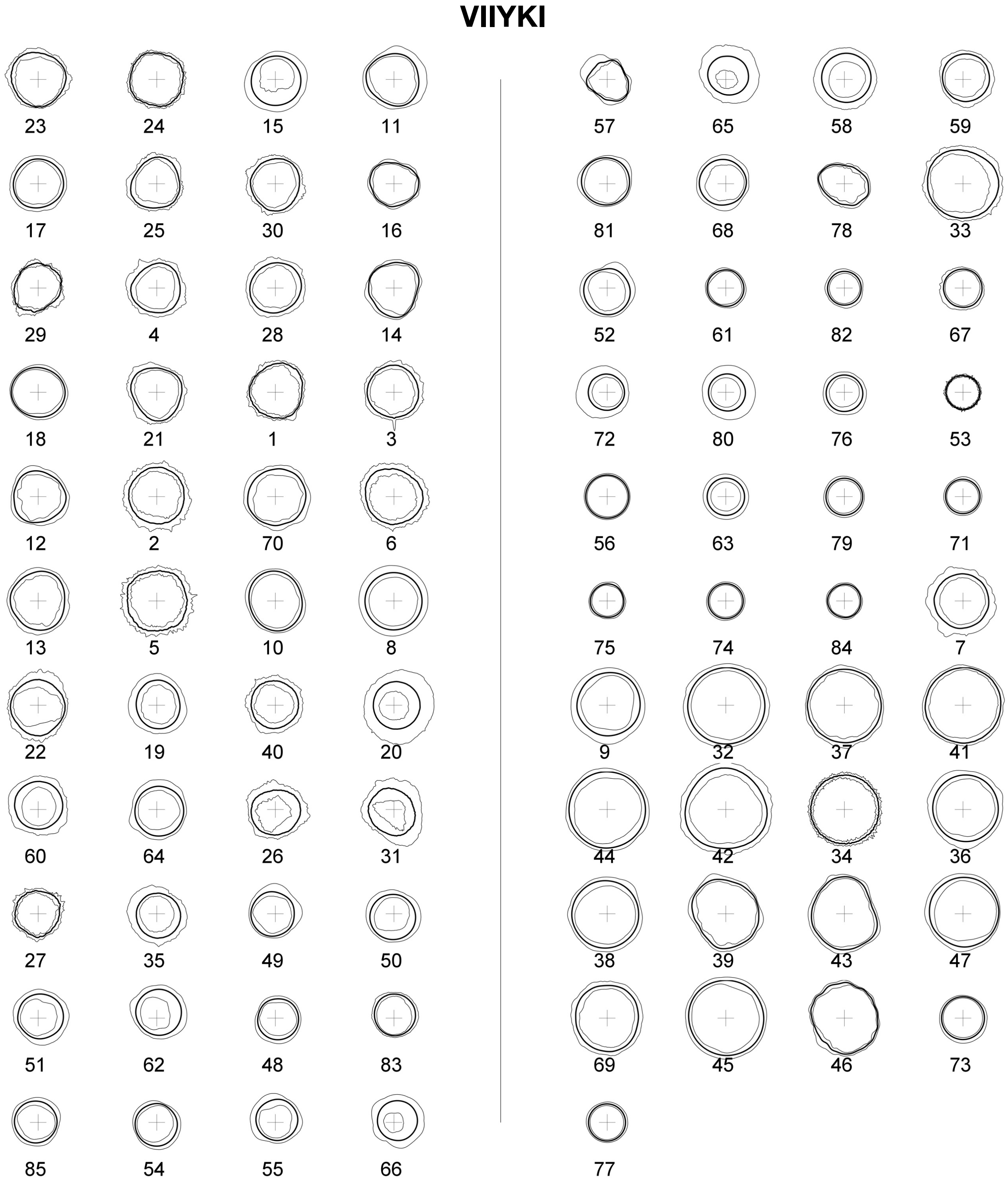

Supplementary Figure 3: Average cross-sectional areas of all 3D models of the Waltz peptide assemblies reconstructed from the AFM data sets. For each fibril 3D model shown in Supplementary Figure SI 3, the average cross-section for that individual fibril is shown as thick solid line. The thin solid lines inside and outside the average cross-sections represent the

minimum and the maximum cross-sections, respectively. They reflect the variations observed in the fibril cross-sections. All of the models are shown with identical scale. The centre cross in each of the model cross-section represent their screw axis, and the length of the horizontal and vertical lines of the cross represent the length of 4 nm. The model cross-sections are shown with their individual index numbers used throughout (see **Supplementary Data**), are arranged by similarity (see **Figure 7** and **Supplementary Figure 6**), and placed in the same order as **Supplementary Figure 1**.

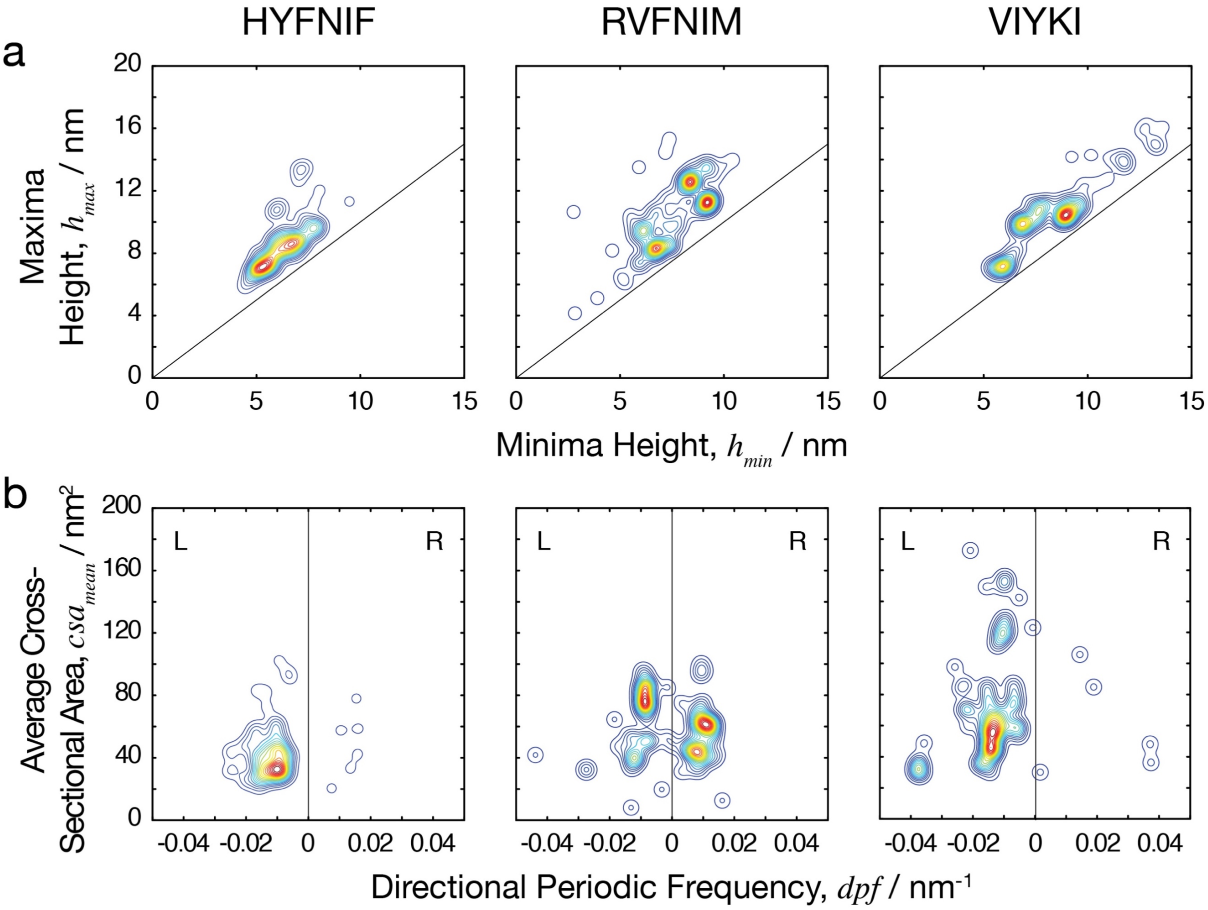

Supplementary Figure 4: The heterogeneity of the Waltz peptide assemblies. (a) The maximum height of the fibrils plotted against the minimum height of the fibrils. (b) The average cross-sectional area of the fibrils plotted against the number of repeating units per nm, with negative and positive values to distinguish handedness (directional periodic frequency, *dpf*). The data is represented as a 2D histogram and visualised as a contour map, where the colouring represents the density of the data-points.

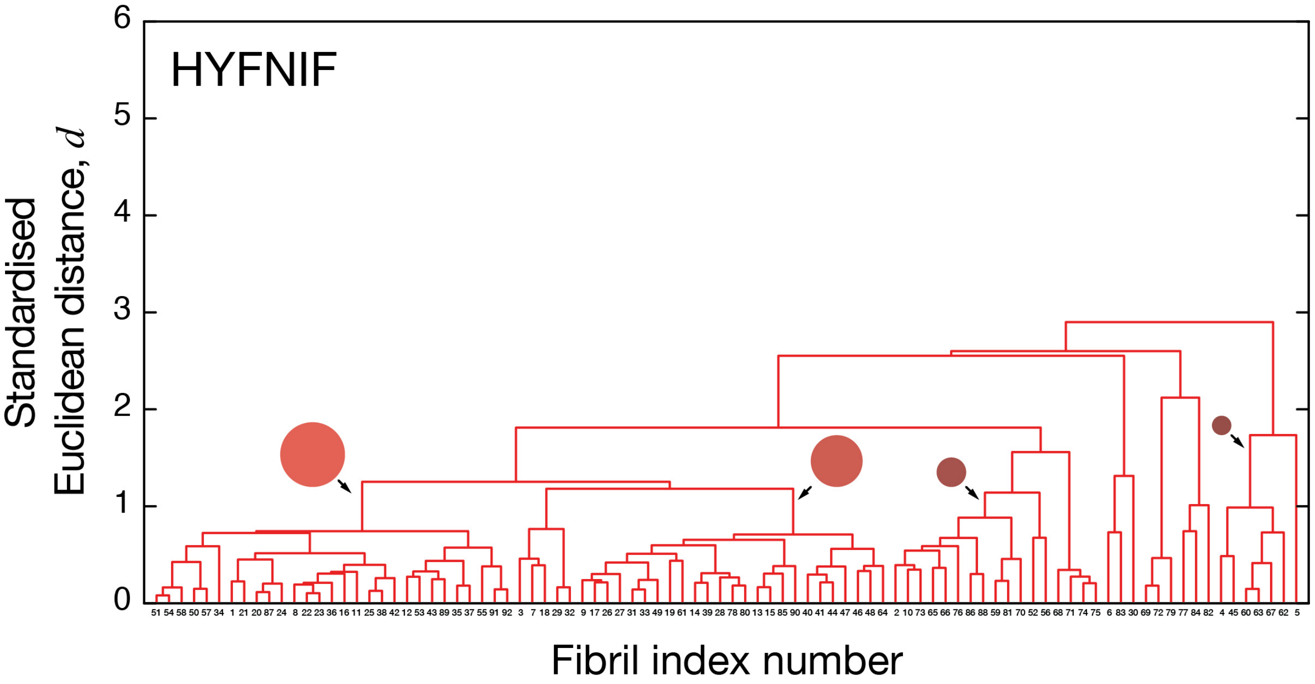
HYFNIF
Standardised Euclidean distance, d
6
5
4
3
2
1
0
Fibril index number

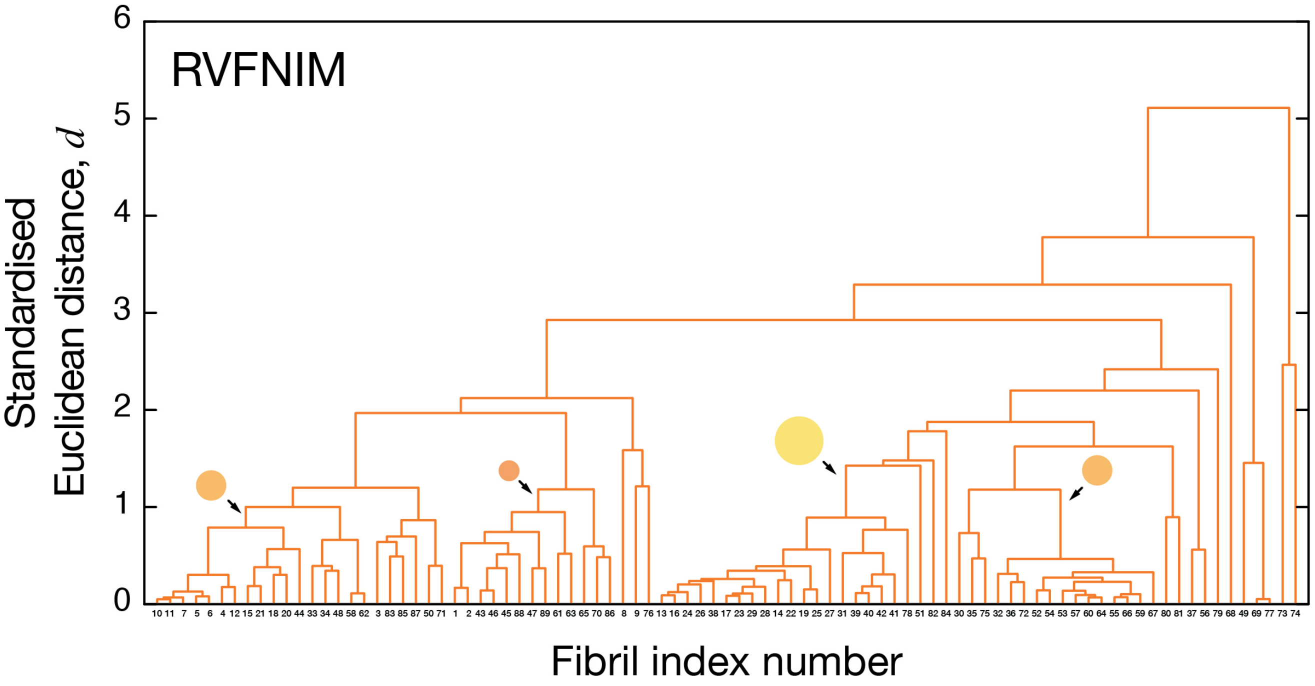
RVFNIM
Standardised Euclidean distance, d
6
5
4
3
2
1
0
Fibril index number

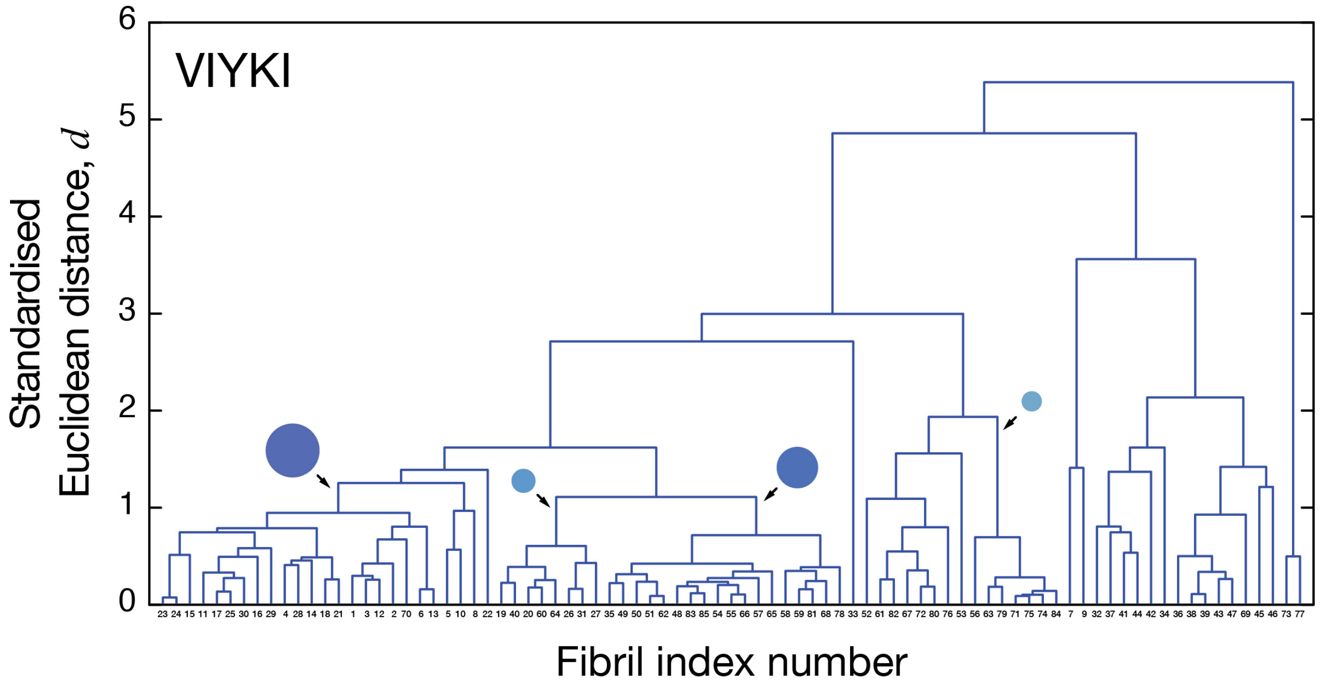

Supplementary Figure 5: Analysis of structural similarity and objective classification of fibril assemblies by agglomerative hierarchical clustering. Full dendrograms representing the full hierarchical relationship between each individual fibril is shown. For each fibril, the individual numbers shown on the x-axis are the fibril index numbers used throughout (see **Supplementary Data**). The x-axis represents the order in which structurally similar fibrils were grouped together, and represent the same order used in **Supplementary Figures 1, 2 and 3**. The coloured circles represent the four largest class for each sample as shown in **Figure 7**.

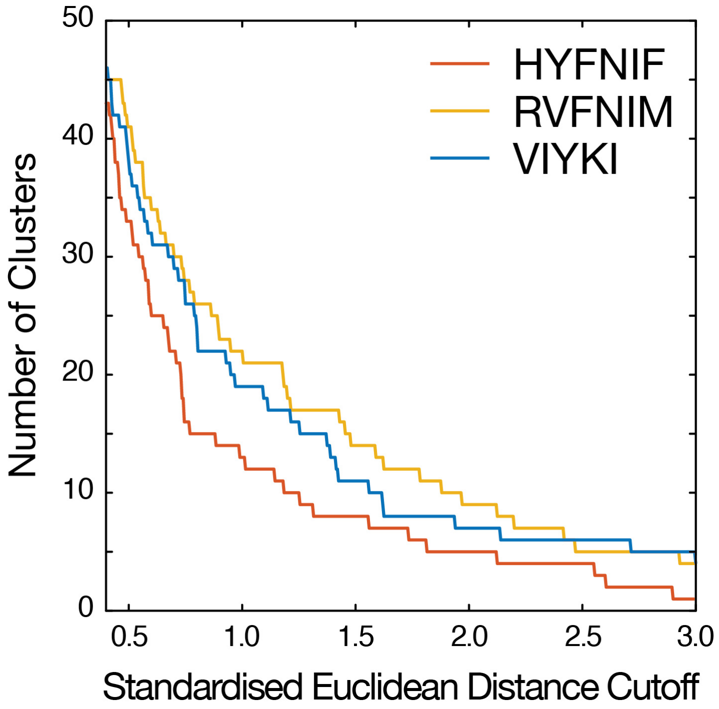

Supplementary Figure 6: The number of clusters generated by agglomerative hierarchical clustering as function of standardised Euclidean distance cut-off used. A standardised Euclidean distance cut-off of 1.0 was used to classify the fibrils in each data set (**Figure 7**). HYFNIF fibrils show greatest similarity as measured by the standard deviation of standardised Euclidean distances, and the data set can be described by smallest number of clusters compared to RVFNIM and VIYKI fibrils, as expected. Despite the standard deviation of standardised Euclidean distance for VIYKI fibrils is greatest of the three samples, roughly the same number of clusters if not more are required to describe the entire RVFNIM data set. This suggest that despite the VIYKI data set showing an overall greater variation, the RVFNIM fibril structures is spread across smaller distances but more evenly compared to VIYKI fibrils.
