# Supplementary material for: Quantification of amyloid fibril polymorphism by nano-morphometry reveals the individuality of filament assembly
Source: Commun Chem. 2020 Sep 11;3:125. doi: 10.1038/s42004-020-00372-3 (PMC9814634; doi:10.1038/s42004-020-00372-3)
Supplement: Supplementary file 2 — Description of Additional Supplementary Files [file 42004_2020_372_MOESM2_ESM.pdf]

Description of Additional Supplementary Files

File Name: Supplementary Data 1
Description: Morphometric parameters for individual fibrils assembled from the peptides HYFNIF, RVFNIM and VIYKI. The contour length of the section of each fibril used to estimate their morphometric parameters and the estimated radius of the cantilever tip used to image each fibril are also shown for each individual fibril. The fibril number is the index number of each of the individual fibrils and were used throughout.
