# Supplementary material for: Quantification of amyloid fibril polymorphism by nano-morphometry reveals the individuality of filament assembly
Source: Commun Chem. 2020 Sep 11;3:125. doi: 10.1038/s42004-020-00372-3 (PMC9814634; doi:10.1038/s42004-020-00372-3)
Supplement: Supplementary file 3 — Supplementary Data 1 [file 42004_2020_372_MOESM3_ESM.pdf]

Quantification of amyloid fibril polymorphism by nano-morphometry reveals the individuality of filament assembly

Supplementary Data

Liam D. Aubrey [1†], Ben J. F. Blakeman [1†], Liisa Lutter [1], Christopher J. Serpell [2], Mick F. Tuite [1], Louise C. Serpell [3], Wei-Feng Xue [1*]

[1] Kent Fungal Group, School of Biosciences, University of Kent, CT2 7NJ, Canterbury, UK

[2] School of Physical Sciences, University of Kent, CT2 7NH, Canterbury, UK

[3] Sussex Neuroscience, School of Life Sciences, University of Sussex, BN1 9QG, Falmer, Brighton, UK

[†] Authors contributed equally to this study

* Correspondence to: W.F.Xue@kent.ac.uk; Tel +44-(0)1227 824821

**Fibril number**	**Length / nm**	**Estimated Tip-Radius / nm**	**Average Height, h_{mean} / nm**	**Directional Periodic Frequency, dpf / nm^{-1}**	**Minimum Height, h_{min} / nm**	**Maximum Height, h_{max} / nm**	**Cross-Sectional Area, csa / nm^2**
			**HYFNIF**				
**1**	4233.6	3.3	6.38	-0.01133	5.06	7.97	30.15
**2**	2919.0	3.1	8.76	-0.01882	7.78	9.82	50.60
**3**	6382.6	3.5	7.88	-0.02459	7.08	8.62	44.58
**4**	6230.5	7.9	9.58	-0.01011	7.79	11.18	62.38
**5**	6884.2	8.0	10.46	-0.01351	9.48	11.32	82.23
**6**	1184.8	6.8	6.51	0.01348	5.74	7.23	33.06
**7**	1800.4	11.8	7.41	-0.02497	6.69	7.94	42.87
**8**	863.6	8.7	6.29	-0.01387	5.52	7.21	29.80
**9**	2224.9	7.3	7.42	-0.00943	6.69	8.33	45.76
**10**	2222.0	11.2	9.00	-0.01664	8.10	9.81	58.98
**11**	1604.3	12.5	6.39	-0.00996	5.36	7.04	31.87
**12**	2353.7	12.7	6.11	-0.01401	5.28	7.01	25.41
**13**	2708.0	9.3	6.94	-0.01181	6.05	8.15	42.06
**14**	1785.8	12.1	7.05	-0.00951	6.15	8.17	33.87
**15**	1294.0	9.8	6.96	-0.01157	6.05	8.26	46.20
**16**	1056.8	11.4	6.51	-0.01133	5.87	7.12	34.55
**17**	629.4	8.7	7.25	-0.00950	6.42	8.34	41.34
**18**	951.5	12.1	7.50	-0.02098	6.95	7.97	47.67
**19**	1074.4	10.5	7.38	-0.00743	6.25	9.12	42.75
**20**	3275.9	12.9	6.56	-0.00824	5.62	7.82	30.91
**21**	2470.9	14.0	6.25	-0.00970	5.12	7.89	25.79
**22**	1185.7	12.1	6.41	-0.01263	5.64	7.32	31.38
**23**	2775.3	13.3	6.51	-0.01224	5.70	7.48	30.63
**24**	3893.7	12.1	6.78	-0.00821	5.68	7.97	33.35
**25**	2227.9	12.0	6.34	-0.01569	5.47	7.28	28.42
**26**	4243.1	11.4	7.45	-0.00801	6.67	8.41	40.46
**27**	6256.3	11.7	7.13	-0.00831	6.44	8.07	37.42
**28**	1362.6	12.8	6.89	-0.01025	5.84	8.66	28.81
**29**	389.7	2.3	7.08	-0.02556	6.10	7.94	31.07
**30**	799.8	5.8	5.18	0.00749	4.40	5.79	20.46
**31**	1669.9	2.0	7.63	-0.00957	6.76	8.54	33.77
**32**	796.9	2.4	7.14	-0.02380	6.06	8.00	33.06
**33**	896.5	1.9	7.69	-0.01002	6.92	8.76	34.53
**34**	1037.1	1.1	6.04	-0.01925	5.15	7.00	46.76
**35**	1869.3	5.6	5.94	-0.01977	5.15	6.85	24.15
**36**	914.1	4.7	6.33	-0.01201	5.54	7.40	26.40
**37**	981.5	4.0	6.05	-0.02034	4.98	7.02	27.18
**38**	2088.9	3.3	6.32	-0.01722	5.44	7.24	27.40
**39**	1517.6	5.0	7.11	-0.00987	6.11	8.46	29.59
**40**	2504.9	6.7	7.44	-0.01835	6.43	8.24	38.93
**41**	4174.8	7.3	7.72	-0.01652	6.75	8.56	39.17
**42**	1391.6	5.6	6.50	-0.01506	5.56	7.67	25.61
**43**	999.0	3.9	5.90	-0.01598	4.98	6.78	21.07
**44**	2062.5	4.5	7.52	-0.01598	6.51	8.62	35.99
**45**	2042.0	5.7	9.92	-0.00978	8.06	12.06	57.88
**46**	533.2	7.5	7.38	-0.01121	6.14	8.97	33.31

**47**	1880.9	4.1	7.85	-0.01647	6.58	9.00	43.65
**48**	612.3	5.6	7.68	-0.01464	6.15	9.16	35.66
**49**	1432.6	5.9	7.49	-0.01115	6.50	8.64	34.90
**50**	3155.3	10.9	6.22	-0.00950	5.40	7.01	41.08
**51**	3638.7	12.4	6.10	-0.01373	5.10	7.16	44.01
**52**	5622.1	13.1	8.85	-0.01671	7.84	9.65	83.03
**53**	1286.1	9.0	6.14	-0.01474	5.10	7.10	31.87
**54**	726.6	13.0	6.07	-0.01371	5.12	7.07	42.14
**55**	3747.1	12.5	5.44	-0.01520	4.61	6.21	40.52
**56**	3295.9	11.5	8.37	-0.01334	7.25	9.19	72.00
**57**	5525.4	11.4	6.23	-0.00977	5.49	7.07	44.66
**58**	609.4	11.7	5.96	-0.01471	5.00	7.01	40.42
**59**	1936.5	5.6	8.39	-0.00619	7.41	10.03	40.26
**60**	665.1	8.8	10.38	-0.01198	7.05	13.37	54.66
**61**	934.6	11.2	7.97	-0.00854	6.70	8.95	46.55
**62**	1834.0	5.7	9.53	-0.00980	7.02	12.61	45.83
**63**	931.8	4.8	10.22	-0.01071	7.11	13.34	52.96
**64**	1711.0	3.8	7.88	-0.01226	6.47	9.58	36.15
**65**	1110.4	3.4	8.73	-0.01348	7.86	9.79	48.84
**66**	855.5	3.2	8.40	-0.01283	7.57	9.22	46.21
**67**	3890.2	10.9	10.64	-0.01105	7.40	13.52	62.20
**68**	2042.9	17.9	7.81	-0.00636	6.03	10.78	37.92
**69**	2077.8	20.0	7.77	-0.00577	7.05	8.78	91.02
**70**	1281.1	11.6	8.33	-0.00857	7.42	9.73	50.56
**71**	2212.1	9.9	8.00	-0.00677	6.18	11.03	28.84
**72**	1666.4	19.1	7.83	-0.00659	7.16	8.83	95.11
**73**	1748.1	8.6	8.55	-0.01657	7.79	9.42	57.09
**74**	1339.5	14.5	7.90	-0.00820	5.80	10.53	28.90
**75**	1307.4	11.5	7.87	-0.00687	5.93	10.79	31.94
**76**	1181.9	9.1	8.76	-0.01013	7.90	9.60	59.36
**77**	2320.0	8.3	8.69	0.01033	7.80	9.45	57.57
**78**	3814.2	8.2	7.24	-0.00996	5.87	8.86	33.87
**79**	1476.7	19.5	7.94	-0.00946	7.20	8.52	102.43
**80**	1333.7	9.8	7.05	-0.00823	5.77	8.73	33.55
**81**	1109.0	13.2	8.47	-0.00630	7.22	10.39	43.35
**82**	1684.6	17.8	7.74	0.01542	6.65	8.67	77.95
**83**	2959.0	19.7	5.88	0.01587	4.86	6.90	42.55
**84**	1801.8	11.1	8.04	0.01608	7.04	8.91	58.71
**85**	6237.4	13.2	6.70	-0.00962	5.83	7.82	43.78
**86**	3014.7	11.2	8.24	-0.01525	6.92	9.06	51.49
**87**	2891.7	12.0	6.51	-0.00864	5.74	7.65	29.87
**88**	1919.0	12.4	8.24	-0.01770	7.24	8.91	54.99
**89**	1781.3	14.7	5.87	-0.01570	4.98	6.84	27.54
**90**	1696.3	22.6	6.81	-0.00883	5.87	8.17	51.99
**91**	1505.9	21.1	5.71	-0.01592	4.79	6.53	31.82
**92**	1330.1	22.3	5.67	-0.01426	4.72	6.46	32.44

**RVFNIM**							
**1**	3317.7	20.6	7.75	-0.01295	6.08	9.25	53.59
**2**	2101.9	13.8	7.98	-0.01331	6.15	9.50	52.72
**3**	599.2	13.6	7.65	0.00831	6.23	9.42	45.05
**4**	1026.0	22.0	7.53	0.01265	6.81	8.42	55.26
**5**	1335.8	23.3	7.48	0.01270	6.76	8.44	58.52
**6**	1034.8	23.6	7.50	0.01254	6.72	8.37	60.36

**7**	1368.0	21.4	7.51	0.01313	6.83	8.36	61.64
**8**	838.9	12.0	7.39	-0.00171	4.62	8.17	53.15
**9**	780.5	27.9	5.43	-0.00639	5.25	6.10	50.30
**10**	2265.4	20.6	7.53	0.01235	6.83	8.37	62.36
**11**	1961.4	20.6	7.54	0.01273	6.78	8.38	63.12
**12**	1178.0	14.6	7.68	0.01356	6.95	8.47	52.26
**13**	945.3	9.4	10.56	-0.00845	8.25	12.43	77.25
**14**	2370.6	9.2	10.25	-0.00885	8.10	12.09	74.65
**15**	1186.8	9.7	7.31	0.01430	6.49	8.23	42.41
**16**	2632.4	8.9	10.64	-0.00873	8.33	12.38	78.72
**17**	4683.1	9.6	10.61	-0.00832	8.47	12.51	73.51
**18**	619.6	9.8	7.12	0.00965	6.45	8.14	39.40
**19**	4566.4	10.5	10.73	-0.00860	8.38	12.82	84.25
**20**	802.6	10.1	7.17	0.00995	6.46	8.06	47.28
**21**	3126.0	9.9	7.30	0.01311	6.63	8.15	38.89
**22**	1813.5	8.0	10.47	-0.00826	8.37	12.19	70.84
**23**	3011.8	8.2	10.69	-0.00863	8.57	12.58	74.25
**24**	1573.3	9.5	10.67	-0.00826	8.32	12.51	75.44
**25**	1579.1	10.7	10.76	-0.00886	8.38	12.49	83.57
**26**	3791.1	8.5	10.75	-0.00844	8.46	12.76	78.81
**27**	1652.4	7.3	10.31	-0.00786	7.94	12.54	64.46
**28**	2179.7	7.1	10.54	-0.00917	8.47	12.69	69.99
**29**	1174.8	7.1	10.73	-0.00850	8.53	12.74	72.98
**30**	952.2	8.6	9.54	0.00036	7.45	11.69	47.97
**31**	1210.0	16.4	11.02	-0.00990	8.51	13.49	82.12
**32**	3410.2	6.0	10.24	0.00703	9.25	11.47	68.18
**33**	1113.3	6.0	8.33	0.00628	7.25	9.57	45.06
**34**	1078.1	6.4	8.48	0.01018	7.36	9.84	40.70
**35**	3020.5	8.3	9.53	0.00629	7.80	11.41	52.48
**36**	1388.7	8.6	10.45	0.00862	9.22	11.62	73.12
**37**	1324.3	12.0	9.76	0.00452	7.17	14.43	46.67
**38**	1505.9	16.7	10.67	-0.00929	8.15	12.92	77.57
**39**	3087.2	8.1	11.55	-0.00777	9.30	13.68	86.29
**40**	1765.3	6.8	11.58	-0.00792	9.10	13.68	86.69
**41**	922.3	11.6	11.39	-0.00866	9.10	13.32	92.91
**42**	4920.1	10.3	11.36	-0.00792	9.17	13.38	86.04
**43**	1809.3	15.9	7.88	-0.01160	6.19	9.72	40.10
**44**	4380.3	13.8	6.80	0.00890	6.06	7.36	43.37
**45**	3154.7	13.7	8.24	-0.01109	6.54	10.06	38.83
**46**	1537.9	12.4	7.83	-0.01234	6.10	9.50	38.63
**47**	2909.5	10.7	7.19	-0.01305	5.68	9.12	33.28
**48**	4561.5	5.1	8.51	0.01030	7.63	9.50	47.76
**49**	4450.2	8.1	7.28	-0.04380	6.68	7.83	41.83
**50**	6172.9	3.5	8.53	0.00939	6.75	10.79	40.14
**51**	6750.0	3.5	12.14	-0.00933	10.43	13.98	86.67
**52**	668.0	3.7	10.28	0.00895	9.47	11.50	60.32
**53**	5168.0	4.1	10.08	0.01006	9.23	11.23	62.88
**54**	3966.8	4.3	10.17	0.00857	9.28	11.35	59.71
**55**	1016.6	4.4	10.09	0.00982	9.18	11.20	67.10
**56**	3366.3	7.4	10.38	0.00624	7.38	15.34	43.17
**57**	2437.5	4.0	10.01	0.00984	9.19	11.18	60.20
**58**	3799.8	4.2	7.78	0.00605	7.33	8.55	42.93
**59**	10898.5	2.4	10.01	0.01000	9.17	11.14	65.11
**60**	1628.9	4.2	9.98	0.01104	9.17	11.21	59.07

**61**	738.3	2.6	7.81	-0.01079	7.48	8.55	45.57
**62**	963.9	2.5	7.83	0.00621	7.39	8.52	45.64
**63**	3293.0	2.5	7.94	-0.01730	7.39	8.56	47.36
**64**	1292.0	4.3	9.98	0.01081	9.18	11.14	60.61
**65**	1895.5	3.6	8.91	-0.00896	7.75	10.18	49.54
**66**	1558.6	4.9	10.11	0.00897	9.19	11.19	66.45
**67**	6820.3	4.5	9.87	0.00718	8.99	10.96	62.40
**68**	5302.2	6.0	9.86	0.00324	2.77	10.65	74.72
**69**	1199.3	5.5	6.62	-0.02746	5.88	7.32	32.52
**70**	1733.4	7.8	9.14	-0.00865	7.14	11.39	52.15
**71**	1277.4	5.6	8.16	0.00703	6.50	10.53	33.97
**72**	5956.1	4.3	10.18	0.01007	9.21	11.38	75.36
**73**	530.3	15.2	3.34	-0.01315	2.83	4.15	8.11
**74**	865.8	7.3	4.33	0.01614	3.91	5.12	12.65
**75**	5804.1	6.8	9.17	0.00964	7.86	10.65	51.21
**76**	3022.0	9.4	5.74	-0.00331	5.05	6.54	19.74
**77**	797.7	8.7	6.67	-0.02747	5.86	7.42	32.31
**78**	6887.7	7.2	10.92	-0.00145	9.55	12.82	85.12
**79**	3433.6	11.8	10.41	-0.01847	5.91	13.51	64.64
**80**	16708.1	15.3	9.84	0.00934	8.89	10.74	100.25
**81**	1790.1	18.3	8.86	0.01005	7.90	9.74	95.32
**82**	7555.8	17.8	9.40	-0.00939	8.38	10.64	95.63
**83**	2493.2	9.8	7.46	0.00801	5.51	10.43	42.50
**84**	5311.6	7.9	11.66	0.00903	9.90	13.50	93.44
**85**	2159.2	8.1	7.30	0.00879	5.62	9.85	31.94
**86**	4060.6	10.8	8.56	-0.00763	6.96	10.59	51.36
**87**	1928.1	12.9	7.27	0.00311	6.15	9.40	31.21
**88**	949.2	13.6	8.32	-0.01367	6.99	9.84	43.15
**89**	6758.9	11.1	7.24	-0.01257	6.06	8.72	39.55
				**VIYKI**			
**1**	1306.7	12.8	9.56	-0.00687	8.61	10.77	75.36
**2**	3902.4	14.1	9.63	-0.00666	8.80	10.61	83.29
**3**	2768.6	8.5	9.82	-0.00650	9.04	10.60	72.35
**4**	3770.5	6.1	9.76	-0.02147	8.99	10.48	59.62
**5**	2941.4	13.3	9.45	-0.02581	8.67	10.18	97.81
**6**	5411.1	12.2	9.64	-0.01515	8.86	10.38	83.65
**7**	3014.7	15.3	9.91	0.01890	9.26	11.03	84.79
**8**	3969.8	4.8	10.49	-0.02241	9.82	11.15	86.68
**9**	1869.2	11.9	11.25	0.01443	10.37	12.40	105.72
**10**	1460.5	9.9	9.54	-0.02392	8.86	10.14	83.66
**11**	3952.3	7.7	9.67	-0.01416	8.93	10.29	72.27
**12**	1983.5	9.1	9.60	-0.00806	8.76	10.36	70.86
**13**	6403.2	11.3	9.48	-0.01561	8.72	10.16	84.75
**14**	872.8	8.8	9.62	-0.02173	8.80	10.25	71.63
**15**	2708.9	8.9	10.50	-0.01365	9.69	11.72	66.02
**16**	814.1	5.4	9.69	-0.01348	9.02	10.38	55.49
**17**	5558.3	2.5	9.69	-0.01367	9.03	10.33	64.84
**18**	951.8	4.8	10.22	-0.02307	9.62	10.87	70.11
**19**	6946.5	2.2	9.46	-0.00648	7.66	10.76	57.19
**20**	3951.3	6.6	9.49	-0.01012	7.82	11.09	59.94
**21**	4168.0	7.7	10.03	-0.02134	9.29	10.68	69.67
**22**	4050.8	8.5	11.01	-0.00642	9.76	12.43	77.84
**23**	2574.6	7.5	10.38	-0.01513	9.57	11.10	76.78

**24**	1801.3	7.6	10.31	-0.01497	9.61	11.00	75.82
**25**	3488.4	10.2	9.80	-0.01261	9.08	10.51	65.85
**26**	2562.4	8.5	9.87	-0.00546	8.01	11.01	60.63
**27**	1144.3	10.8	9.74	-0.00523	8.66	11.30	53.32
**28**	3482.5	10.3	9.87	-0.01808	9.16	10.53	67.28
**29**	704.7	11.7	10.02	-0.01131	9.07	11.40	58.51
**30**	3307.8	9.5	9.88	-0.01058	9.16	10.68	68.44
**31**	2402.2	8.5	9.97	-0.00499	8.13	11.25	58.61
**32**	5220.7	5.3	14.82	-0.00996	13.65	16.08	155.04
**33**	5677.8	5.4	9.46	-0.00951	7.19	11.27	128.53
**34**	12767.6	5.3	14.13	-0.00070	12.86	16.25	123.08
**35**	1895.5	5.3	8.30	-0.01212	6.54	9.95	52.77
**36**	4488.3	5.9	12.70	-0.01158	11.09	14.07	114.90
**37**	3580.1	5.2	14.05	-0.00503	13.08	15.21	142.31
**38**	3240.2	3.0	12.99	-0.01018	11.64	14.18	118.56
**39**	2050.8	2.9	12.90	-0.01023	11.71	13.97	118.45
**40**	1957.0	4.7	9.12	-0.00766	7.60	10.75	59.94
**41**	1716.8	5.8	14.19	-0.01571	13.44	14.92	149.40
**42**	5619.2	9.0	14.10	-0.02081	12.58	15.80	172.76
**43**	1804.8	4.8	12.82	-0.01162	11.76	13.53	121.96
**44**	3958.0	9.6	14.02	-0.00934	13.29	14.81	153.36
**45**	2836.0	9.0	12.62	-0.01022	10.16	14.29	149.29
**46**	665.0	5.0	12.44	-0.00899	9.21	14.17	120.73
**47**	1198.3	9.3	12.91	-0.00999	11.88	13.78	126.90
**48**	1207.0	3.1	8.47	-0.01488	6.75	9.97	44.71
**49**	770.5	5.7	8.19	-0.01425	6.64	9.71	51.21
**50**	4133.8	5.7	8.68	-0.01427	7.09	10.04	54.75
**51**	2097.7	5.7	8.48	-0.01238	6.89	10.05	54.08
**52**	565.4	7.8	6.08	-0.01585	5.36	7.05	56.46
**53**	4749.0	3.5	6.23	0.00168	5.54	7.13	30.06
**54**	1271.5	2.6	8.55	-0.01491	7.08	9.90	47.08
**55**	823.3	2.9	8.53	-0.01334	6.94	9.98	45.39
**56**	1511.7	1.1	6.27	-0.03569	5.71	6.87	48.89
**57**	477.5	3.3	8.47	-0.01460	7.02	9.45	42.41
**58**	10878.0	12.8	8.80	-0.01562	7.49	9.99	64.85
**59**	2214.9	10.6	8.94	-0.01443	7.59	10.36	59.29
**60**	3012.7	12.2	9.48	-0.01228	7.80	11.06	61.31
**61**	1317.5	11.5	6.73	-0.01591	6.20	7.27	35.32
**62**	1461.0	9.2	8.38	-0.01230	6.84	10.05	55.94
**63**	6555.3	12.1	6.68	-0.03752	6.22	7.37	37.12
**64**	3340.6	8.6	9.34	-0.01017	7.58	10.90	64.45
**65**	5198.4	3.4	8.03	-0.01346	6.63	9.38	44.62
**66**	1679.1	5.2	8.39	-0.01249	6.96	9.96	44.54
**67**	2332.5	2.8	7.31	-0.01413	6.72	8.33	38.99
**68**	2124.5	4.9	9.04	-0.01411	7.53	10.67	56.58
**69**	7116.3	16.1	11.82	-0.01222	10.97	12.74	111.69
**70**	2097.7	16.7	10.19	-0.00714	9.14	11.36	86.84
**71**	1333.0	4.3	6.46	-0.03744	5.88	7.18	31.12
**72**	2097.7	4.4	6.94	-0.01762	6.35	7.99	35.76
**73**	1048.9	4.5	6.42	0.03711	5.95	7.13	48.25
**74**	1145.5	4.6	6.51	-0.03834	5.96	7.14	32.16
**75**	2774.4	4.6	6.46	-0.03781	5.96	7.08	29.69
**76**	5845.3	11.1	6.16	-0.01778	5.36	7.13	35.51
**77**	1549.6	7.2	6.24	0.03739	5.74	6.86	36.13

**78**	846.7	5.3	8.81	-0.01532	7.47	10.10	51.79
**79**	1974.6	6.6	6.52	-0.03693	5.97	7.29	35.61
**80**	6820.4	4.2	7.11	-0.01583	6.43	8.04	37.61
**81**	1541.0	5.1	9.04	-0.01556	7.76	10.20	60.05
**82**	2862.3	5.0	6.61	-0.01431	6.04	7.34	30.01
**83**	3518.6	5.8	8.20	-0.01449	6.70	9.66	45.13
**84**	2493.2	5.0	6.50	-0.03646	5.95	7.11	29.02
**85**	1095.7	5.5	8.30	-0.01457	6.84	9.69	47.00
